# Interspecies differences in protein expression do not impact the spatiotemporal regulation of glycoprotein VI mediated activation

**DOI:** 10.1111/jth.14673

**Published:** 2019-12-06

**Authors:** Joanne L. Dunster, Amanda J. Unsworth, Alexander P. Bye, Elizabeth J. Haining, Marcin A. Sowa, Ying Di, Tanya Sage, Chiara Pallini, Jeremy A. Pike, Alexander T. Hardy, Bernhard Nieswandt, Ángel García, Steve P. Watson, Natalie S. Poulter, Jonathan M. Gibbins, Alice Y. Pollitt

**Affiliations:** ^1^ Institute for Cardiovascular and Metabolic Research (ICMR) School of Biological Sciences University of Reading Reading UK; ^2^ Department of Life Sciences School of Science and Engineering Manchester Metropolitan University Manchester UK; ^3^ Institute of Cardiovascular Sciences (ICVS) College of Medical and Dental Sciences University of Birmingham Birmingham UK; ^4^ Platelet Proteomics Group Center for Research in Molecular Medicine and Chronic Diseases (CIMUS) Universidade de Santiago de Compostela Santiago de Compostela Spain; ^5^ Centre of Membrane Proteins and Receptors (COMPARE) Universities of Birmingham and Nottingham Midlands Birmingham UK; ^6^ Department of Experimental Biomedicine University Hospital University of Würzburg Würzburg Germany

**Keywords:** GPVI, mathematical modelling, platelet, Syk

## Abstract

**Background:**

Accurate protein quantification is a vital prerequisite for generating meaningful predictions when using systems biology approaches, a method that is increasingly being used to unravel the complexities of subcellular interactions and as part of the drug discovery process. Quantitative proteomics, flow cytometry, and western blotting have been extensively used to define human platelet protein copy numbers, yet for mouse platelets, a model widely used for platelet research, evidence is largely limited to a single proteomic dataset in which the total amount of proteins was generally comparatively higher than those found in human platelets.

**Objectives:**

To investigate the functional implications of discrepancies between levels of mouse and human proteins in the glycoprotein VI (GPVI) signalling pathway using a systems pharmacology model of GPVI.

**Methods:**

The protein copy number of mouse platelet receptors was determined using flow cytometry. The Virtual Platelet, a mathematical model of GPVI signalling, was used to determine the consequences of protein copy number differences observed between human and mouse platelets.

**Results and conclusion:**

Despite the small size of mouse platelets compared to human platelets they possessed a greater density of surface receptors alongside a higher concentration of intracellular signalling proteins. Surprisingly the predicted temporal profile of Syk activity was similar in both species with predictions supported experimentally. Super resolution microscopy demonstrates that the spatial distribution of Syk is similar between species, suggesting that the spatial distribution of receptors and signalling molecules in activated platelets, rather than their copy number, is important for signalling pathway regulation.

**Essentials**

- Mouse platelets have strikingly higher copy numbers of some proteins compared to human platelets.
- Functional implications of discrepancies are explored using a systems pharmacology model of GPVI.
- Interspecies differences in protein expression do not impact the regulation of GPVI signalling.
- Regulation of GPVI signalling is spatially regulated at the platelet membrane in humans and mice.

## 1 INTRODUCTION

Platelets are small anucleate cells that play a vital role in vascular integrity and the prevention of excessive bleeding. In addition to this key role in hemostasis inappropriate platelet regulation contributes to cardiovascular and inflammatory diseases. These platelet‐mediated processes involve receptor‐ligand interactions and the initiation of complex signalling cascades. Indeed, current antiplatelet drugs target receptor‐ligand interactions or signalling cascades to reduce cardiovascular events, but also have the side effect of an increased risk of bleeding. There is therefore a pressing need for safer antiplatelet drugs, but the high cost of clinical trials has discouraged drug development. Pharmaceutical companies are increasingly adopting quantitative systems pharmacology (QSP) approaches to determine mechanisms of action of new and existing drugs and to better utilize preclinical data to optimize clinical trial design.^1^, ^2^ QSP benefits greatly from published quantitative data such as receptor and signalling protein expression levels so that theoretical models can generate more accurate predictions.

Glycoprotein VI (GPVI), a receptor for collagen, laminin, and more recently recognized as a receptor for fibrin and fibrinogen, represents an attractive antithrombotic target in experimental models with expression limited to platelets and megakaryocytes.^3^, ^4^, ^5^, ^6^, ^7^, ^8^, ^9^, ^10^, ^11^ Following ligand engagement a signalling cascade is initiated that culminates in platelet activation. While the major components of this pathway are well known the underlying mechanism of activation has not yet been fully elucidated. Antagonists of the platelet collagen receptor GPVI and Bruton tyrosine kinase (Btk) inhibitors (which inhibit signalling evoked by GPVI) are recognized as potential antiplatelet drugs,^12^, ^13^, ^14^, ^15^, ^16^, ^17^, ^18^, ^19^ although copy numbers of receptors and signalling molecules involved in the GPVI signalling pathway vary widely between individuals and even more so between humans and mice.^20^, ^21^, ^22^ The functional consequences of these differences and the implications for the development of drugs that target this pathway are poorly understood.

Quantification of cell protein copy numbers is a critical step in the development of a predictive model of platelet activation.^23^ Quantitative proteomics, flow cytometry, and western blotting have been used to measure human platelet protein copy numbers.^24^, ^25^, ^26^, ^27^ Most published reports of platelet protein copy numbers have been in humans and tend to focus on a single protein of interest. No previous study has provided a systematic comparison between different quantification methods or between species (human and mouse). Zeiler et al^28^ published the mouse platelet proteome by exploiting quantitative proteomics and reported strikingly higher copy numbers of some proteins in mouse compared to human platelets, some an order of magnitude higher in mice than humans. This was especially surprising since mouse platelets are approximately half the volume of human platelets (4.3 versus 7.4 fL)^29^, ^30^ implying that the densities of these proteins within mouse platelets are higher. Few studies of mouse protein levels in platelets exist to corroborate these surprising findings. One such study quantified mouse Src family kinases using western blotting to compare the signal intensity of a platelet lysate with known amounts of recombinant protein.^31^ The copy number of Src corroborated well with the Zeiler et al(28) proteomic dataset but the copy numbers of Fgr, Fyn, and Lyn differed by up to 240 orders of magnitude. This may reflect differences in the binding capacity of antibodies for the native protein compared to recombinant protein or technical issues associated with the analysis of lipid modified proteins by mass spectrometry.

We sought to independently determine mouse copy numbers of key platelet proteins using quantitative flow cytometry as an accessible method to complement mass spectrometry. To address the relevance of differences in protein expression we used GPVI as a model receptor. Using a systems pharmacology model of human platelet GPVI signalling, which we call the Virtual Platelet,^29^ the functional implications of discrepancies between levels of mouse and human proteins in the GPVI signalling pathway were explored. The model is a dynamic mathematical model that captures the initial events that occur following GPVI receptor activation. We addressed the spatial, temporal, and functional questions raised by the mathematical model experimentally using mice expressing kinase dead Syk, western blotting for phosphorylated Syk at Y525/526, and super‐resolution microscopy.

## 2 METHODS

### 2.1 Flow cytometry to determine surface protein copy number

Saturating concentrations of fluorescently labelled monoclonal antibodies were applied to beads of known antigen binding capacity (Quantum Simply Cellular; Bangs Laboratories) and 10^6^ washed human and mouse platelets. Platelets were also incubated with fluorescently labelled IgG to control against nonspecific antibody binding. Bead and platelet fluorescence was read using flow cytometry (BD Biosciences; FACSVerse, Accuri CSampler Plus). The geometric mean fluorescence was used to construct a standard curve to enable the protein copy number on the surface of platelets to be determined. A linear regression was fitted to the standard curve. Bead saturation was confirmed by a high R^2^ value close to 1. All monoclonal and FITC conjugated antibodies were from Emfret Analytics (5 µL/10^6^ platelets) except anti‐CLEC‐2 (10 µg/mL INU1); anti‐CD41 (30 µg/mL MWReg30, BD Biosciences) anti‐ADAM10 (10 µL/10^6^ platelets R&D systems); PE conjugated anti‐human GPVI antibody (2.5 µL/10^6^ platelets HY101, BD Pharmingen).

### 2.2 Flow cytometry to determine intracellular protein copy number

8 × 10^6^ human washed platelets suspended in 1× Hepes buffered saline (HBS) were fixed with an equal volume of 4% Paraformaldehyde for 10 minutes at room temperature. Platelets were washed three times with 1× HBS with pelleting for 15 minutes at 500 g. Platelets were permeabilized by incubation with BD Phosflow Perm Buffer III for 30 minutes on ice. Following three washes with 1× phosphate buffered saline, platelets were incubated with a saturating concentration of fluorescein isothiocyanate (FITC) conjugated anti‐human Syk antibody 4D10 or FITC conjugated IgG control for 20 minutes at room temperature (20 µg/mL; BD Pharmingen). The geometric mean fluorescence was compared to beads of known antigen binding capacity as described above to determine the copy number of human Syk.

### 2.3 Virtual platelet predictions

The mathematical model used to generate computational predictions (the Virtual Platelet) has been described previously.^29^ The model captures the interactions between key proteins downstream of collagen receptor GPVI, and simulations form predictions of how the proteins interact, to bind, regulate, and activate over time. Experimental data describing the copy numbers of the proteins GPVI, Syk, c‐Cbl, and Tula‐2 form model inputs and along with estimates of platelet volume, allow numerical solutions of the model (carried out with the numerical solver code of R package deSolve^32^) to predict how variation in protein copy numbers affects signalling downstream of the GPVI receptor.

Full details of the interactions captured in the model, its equations, and methods of calibration and validation are available in Dunster et al(29) and an interactive online interface to the virtual platelet is provide at https://cardiomaths.shinyapps.io/VirtualPlateletInterspecies and the R code is available on request.

Local sensitivity analysis was performed by varying each protein copy number by 50% above and below their initial value, the time to reach peak Syk activity was calculated according to

$$\text{Sensitivity}\;\text{Score}\;=\;\frac{O_{a}-O_{i}}{O_{a}}$$

where *O*_a_ and *O*_i_ represent the time to reach the peak in Syk activity in respect of the initial protein copy numbers.

### 2.4 Human platelet preparation

Human platelets were purified from citrated blood from consenting aspirin‐free, healthy volunteers following procedures approved by the University of Reading Research Ethics Committee and prepared as previously described.^29^

### 2.5 Mouse platelet preparation

Procedures were approved by the University of Reading’s Animal Welfare and Ethical Review Body. Blood was obtained from C57/BL6 mice via cardiac puncture into acid citrate dextrose (ACD) following CO_2_ narcosis and platelets prepared (4 × 10^8^ platelets/mL) as previously described.^33^ Platelet aggregation at 2 × 10^8^ platelets/mL was followed using light transmission aggregometry as previously described.^34^

### 2.6 Syk kinase dead expressing mouse model

Animal experimentation was performed with ethical approval from the UK Home Office (PPL P0E98D513) granted to the University of Birmingham. Syk kinase‐dead (Syk KD) mice refer to the following novel mouse strain: C57BL/6NTac‐*Syk^tm3515(K396R)Arte^* (Taconic Artemis), which expresses a Syk protein with a K396R point mutation in the presence of cre recombinase. For this study, mice were crossed with mice carrying the platelet and megakaryocyte specific Pf4 promoter driven cre. Experiments were performed by blinding the genotypes prior to the experiment and during analysis.

### 2.7 Syk Y525/526 and LAT Y200 phosphorylation time course

Human and mouse washed platelets (4 × 10^8^ cells/mL) were prepared under non‐aggregating conditions with stirring using an AggRAM aggregometer (Helena Bioscience) for the indicated time points before lysis as described previously.^26^ Anti‐phospho Syk (Abcam; ab58575); anti‐phospho LAT (Abcam; ab68139); anti‐Actin antibody (C‐11; Santa Cruz; sc‐1615); anti‐Syk 4D10 (cell signalling).

### 2.8 Platelet spreading and staining

For stochastic optical reconstruction microscopy (STORM) imaging washed human and mouse platelets were spread on C‐reactive protein (CRP) coated 35 mm #1.5 (0.17 mm) glass bottomed dishes (MatTek Corporation, USA) as previously described.^35^ Fixed and permeabilized platelets were labelled with a pan‐Syk antibody (Santa Cruz; N‐19:sc‐1077 used at 1 µg/mL) at room temperature for 1 hour followed by anti‐rabbit‐Alexa647 (Life Technologies; A‐21245 used at 1:300 dilution) secondary labelling and Phalloidin‐Alexa488 (1:300 dilution) at room temperature for 1 hour. Samples were washed and stored in phosphate‐buffered saline (PBS) until imaged.

### 2.9 STORM imaging

Samples were imaged on a Nikon N‐STORM system in dSTORM mode which is characterized by a Ti‐E stand with Perfect Focus, 100 × 1.49 NA TIRF objective lens, Agilent Ultra High Power Dual Output Laser bed (170‐mW, 647‐nm laser) for the excitation and Andor IXON Ultra 897 EMCCD camera for the image acquisition. To allow fluorophore blinking, samples were imaged in a PBS based buffer consisting of enzyme solution (catalase 1 µg/mL, Tris [2‐carboxyelthyl] phosphine hydrochloride 4 mmol/L, glycerol 50%, KCl 25 mmol/L, pH 7.5 Tris‐HCl 20 mmol/L, glucose oxidase 50 µg/mL), glucose solution (glucose 100 mg/mL, glycerin 10%), and reducing agent solution (100 mmol/L MEA). For single color (Alexa647) the N‐STORM emission cube was used and the 405 laser power was then increased by 5% every 30 seconds during imaging to reactivate the fluorophore from the dark state. 20 000 frames were captured using Nikon NIS Elements v4.5 with an exposure time of 20 ms, gain 300, and conversion gain 3, and reconstructed using STORM analysis module 3.2, applying the drift correction and the Gaussian rendering. Five separate fields of view (FOV) from three independent experiments were imaged for both mouse and human platelets. Identified points, which represent individual fluorescent blinking events, were filtered on photon count and only those with a count >500 were selected for further cluster analysis.

### 2.10 Analysis of dSTORM data

After localizing detections (average precision 10 nm) within NIS‐Elements density‐based spatial clustering of applications with noise (DBSCAN)^36^ was used to group detections into clusters. For DBSCAN the radius of the local neighborhood was set to 25 and the minimum number of directly reachable points was set to 10. Edge points were included in clusters. Cluster area was calculated using the convex hull of all detections within a cluster and cluster detection density was defined as the number of detections within a cluster divided by the cluster area. Analysis was performed on whole fields of view and measurements for all clusters within a technical replicate were grouped. This analysis was performed using the R package RSMLM.^37^ To measure cellular area and calculate clusters' per/µm^2^ regions of interest (ROIs) were drawn around the cellular boundary using the epi image within Nikon NIS‐Elements.

### 2.11 Statistical analysis

Data is presented as the mean ± standard deviation. Where indicated statistical analysis was performed using unpaired two‐tailed *t*‐test or 2‐way analysis of variance (ANOVA) with Bonferroni post‐test. All statistical analyses were performed using GraphPad Prism 7.

## 3 RESULTS

### 3.1 Mouse platelets have a greater density GPVI

**Figure 1:**
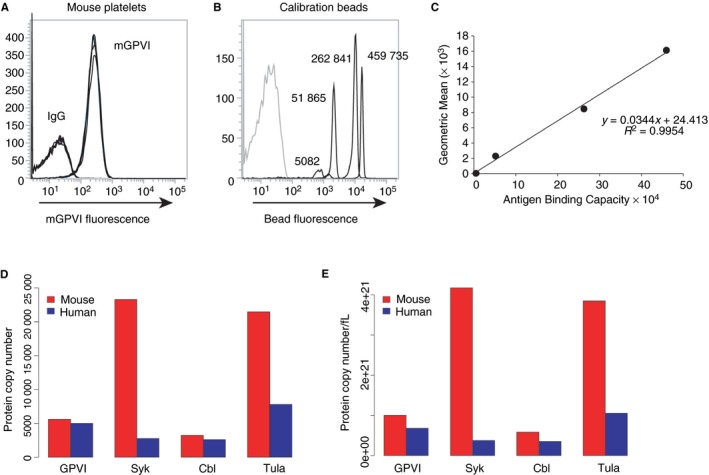
Flow cytometry can be used to determine the copy number of mouse platelet receptors. Saturating concentrations of directly dye conjugated monoclonal antibodies against mouse GPVI (JAQ1) were used to label platelets (A) and beads of known antigen binding capacity (B). The geometric mean was used to construct a calibration graph, which can then be used to calculate the number of proteins exposed at the platelet cell surface (C). Comparison between mouse and human platelet copy numbers taken from this study, Zeiler et al (28) and Mazet et al (26) (D). Comparison between mouse and human protein densities, based on platelet volumes of 4.3 and 7.4 fL for mice and human, respectively (E)

Mouse receptor copy numbers were determined by comparing antibody labelled platelets to antibody labelled calibration beads with known antigen binding capacities. The mean fluorescence intensity of platelets stained with a monoclonal antibody to mouse GPVI (JAQ1) (Figure 1A) were compared to beads of known antigen binding capacity, also labelled with JAQ1 (Figure 1B), which were used to construct a calibration curve (Figure 1C). Using this, mouse platelets were determined to have 5586 ± 1155 copies of GPVI at the cell surface, which is similar to proteomic estimates.

To validate the flow cytometry approach the surface copy number of other membrane proteins was determined and compared to the published mouse proteomic database (Table 1).^28^ Copy number was similar for CLEC‐2, integrins α2, αIIb and α6, GPIbα and P‐selectin (activated) platelets, whereas the levels of CD9 and ADAM10 were approximately one order of magnitude higher when measured using proteomics.

**Table 1:** Copy number of major mouse platelet receptors. Copy number of major mouse platelet receptors determined by flow cytometry values presented as the mean ± S.D n> 3

Mouse platelet copy number (copy number ± copies)		
Platelet receptor	Proteomics^a^	Flow cytometry
GPVI	7822 ± 637	5586 ± 1155
α2	17 591 ± 1260	25 418 ± 4594
CLEC‐2	41 652 ± 7759	42 816 ± 637
CD9	135 059 ± 11 862	10 544 ± 2481
ADAM10	9889 ± 1151	1175 ± 303
P‐selectin	35 970 ± 2712	
P‐selectin (resting)		550 ± 290
P‐selectin (activated)		53 538 ± 8875
αIIb (CD41)	10 6624 ± 7905	142 027 ± 11 782
α6	20 672 ± 1535	11 312 ± 1593
GPIbα	46 154 ± 5106	61 851 ± 26 852
Syk	23 286 ± 4114	
Cbl	3241 ± 416	
Tula2	21 469 ± 3083	

Note

A comparison has been made with the available mouse quantitative proteomics data taken from. 28

a Zeiler et al. 28

The approach was further validated by using the flow cytometry method to determine the surface copy number of human GPVI and the intracellular protein copy number of human Syk and comparing these to the published human proteomic database (Table 2).^24^ The copy number for human Syk was similar, whereas human GPVI was approximately two‐fold higher when measured by flow cytometry compared to the proteomic estimation.

**Table 2:** Copy number of human GPVI and human Syk. Copy number of human GPVI and human Syk determined by flow cytometry, values presented as the mean ± S.D n ≥ 3

Human platelet copy number (copy number ± copies)		
Platelet Protein	Proteomics^a^	Flow cytometry
GPVI	9600^a^	25 384 ± 3639
Syk	4600^a^	5192 ± 1055

Note

A comparison has been made with the available human quantitative proteomics data taken from.^24^

a Burkhart et al.^24^

### 3.2 Modelling of mouse GPVI signalling using the Virtual Platelet simulation

When comparing the published protein copy numbers for proteins involved in the GPVI signalling pathway (GPVI, Syk, Cbl, and TULA‐2) in human and mouse platelets there are some striking differences (Figure 1D).^24^, ^26^, ^28^ One difference of note is the 10‐fold increase in Syk molecules per mouse platelet compared to human platelets. Additionally, due to the smaller size of mouse platelets compared to human platelets, all molecules involved in the initial events downstream of GPVI, including the receptor, are at a greater density in mouse platelets than in human (Figure 1E).

**Figure 2:**
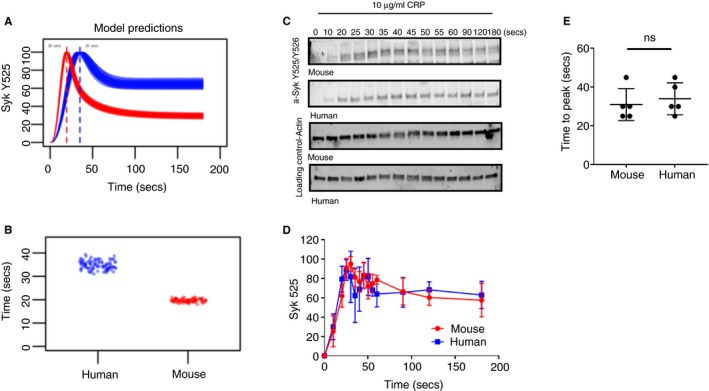
Virtual platelet predictions of Syk activity in a hypothetical population of mouse and human platelets is supported by the experimental outcomes. A, The results of 200 simulations of the Virtual Platelet are shown. Each line represents a prediction of how Syk phosphorylation changes over time in response to protein copy numbers randomly selected across their normal range (Human: GPVI, 5000 +/−12%; Syk, 2763 +/−22%, c‐Cbl, 2581 +/−25%. Mouse: GPVI, 5586 +/−12%; Syk, 23 286 +/−22%, c‐Cbl, 3241 +/− 25%). Simulations representing Syk phosphorylation in a hypothetical mouse platelet (n = 100) shown in red or in a human platelet (n = 100) in blue. B, A summary of model predictions for the time to reach maximal peak Syk activity with each simulation denoted graphically by a circle. C, Representative time course of Syk Y525/Y526 phosphorylation in human and mouse washed platelets following GPVI ligation with 10 µg/mL CRP. Actin acts as a loading control. D, Quantification of mouse and human Syk Y525/Y526 phosphorylation time courses. Mean ± S.D, n = 5. E, Time to maximal Syk Y525/Y526 phosphorylation. Line indicates the mean ± S.D. *P* = .5796 Statistical analysis was performed with unpaired *t* test

The Virtual Platelet model of human GPVI signalling^29^ was used to predict how these large differences in mouse platelet protein copy number influence signalling. The model is able to predict the effects of variability in protein copy number on events downstream of the GPVI receptor. We replaced parameters in the Virtual Platelet model with mouse protein copy numbers to enable comparison between the dynamics of GPVI signalling between the two species (Figure 2A and 2). The model was used to predict the dynamics of Syk tyrosine phosphorylation at positions Y525/Y526 (Y519/520 in mouse). Phosphorylation of Y525/Y526 within the activation loop of the kinase domain of human Syk is a recognized Syk activation marker^38^ and a critical step in GPVI signalling.

Time‐dependent simulations of Syk phosphorylation on the activatory loop (Y525/526) in platelets from a hypothetical population of human and mouse donors following ligation of GPVI with 10 µg/mL CRP display a similar temporal pattern of tyrosine phosphorylation (Figure 2A). The virtual human population show a peak in Syk phosphorylation at the activatory site occurring in the range of 30‐39 seconds following ligand being applied, while the virtual mouse population peaked between 18‐22 seconds (Figure 2B).

### 3.3 Experimental time course of human and mouse Syk activation corroborates the outcomes of the modelling

To corroborate the outcomes of the modelling, experimental time‐courses of Syk phosphorylation at Y525/526 were determined (Figure 2C and 2D). Due to the high sequence similarity between human and mouse Syk, the phospho‐specific antibody raised against phosphorylated tyrosines Y525/526 recognizes the corresponding phosphorylated residues Y519/520 in mouse platelets.^38^ The time to maximal tyrosine phosphorylation on the Syk activatory loop was determined by quantitative western blotting, which for mouse was 31 ± 8 seconds and for human was 34 ± 8 seconds following stimulation with 10 µg/mL CRP (Figure 2E), similar to the times to peak predicted by the Virtual Platelet model.

### 3.4 Mouse platelets are refractory to large reductions in the number of Syk molecules

**Figure 3:**
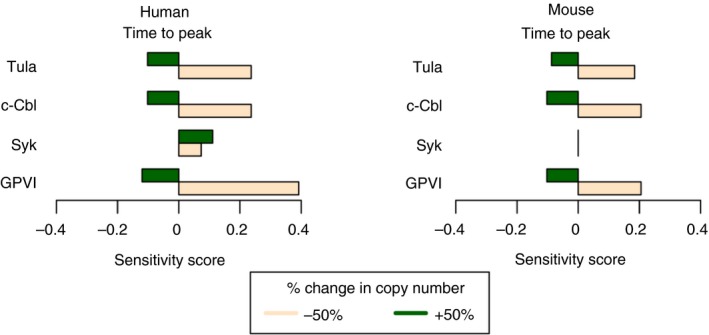
Mouse platelets are refractory to large reductions in the number of Syk molecules. Modelling suggests that the sensitivity of Syk phosphorylation to variation in protein copy numbers varies between human and mouse platelets. Time to maximal Syk phosphorylation in a hypothetical population of human and mouse platelets is shown, where the copy number of each protein was varied by 50% above and below their initial value. A positive sensitivity score represents an increase in the time to reach peak activity and a negative score represents a decrease

**Figure 4:**
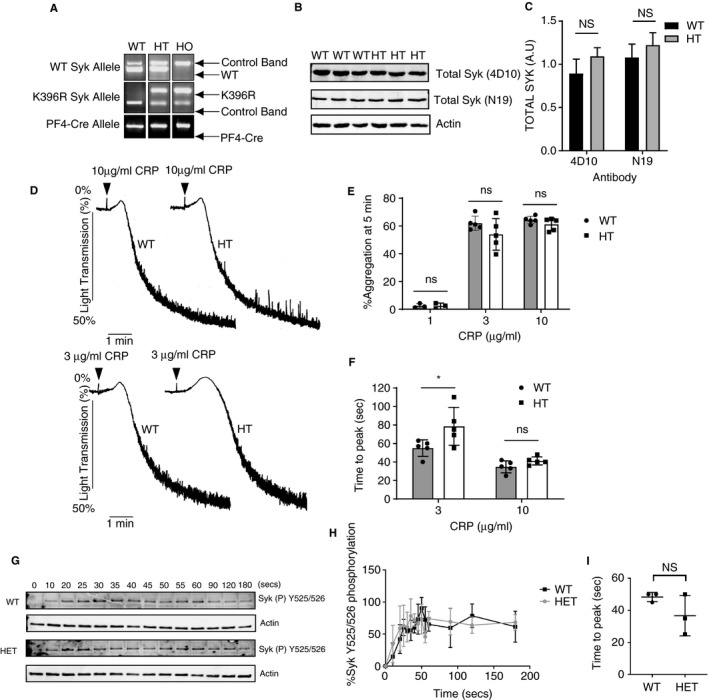
A, Genotyping strategy confirming the presence of both a Syk wild‐type (WT) allele and kinase dead (KD) version under the control of PF4‐Cre in the heterozygous mice (HT) and homozygous mice (HO). B, Total Syk protein levels were measured by western blot in control (WT) and Syk KD HT (HT) mice using two different anti‐Syk antibodies. C, Quantification of Syk levels in platelets from WT and Syk KD HT mice. Mean ± S.D, n = 3. 4D10 *P* = .146, N19 *P* = .305 Statistical analysis was performed with unpaired *t* test. D, Platelet aggregation of washed control (WT) and heterozygous (HT) mice induced by 10 and 3 µg/mL CRP (addition of agonist indicated by arrowhead). E, Quantification of % aggregation at 5 minutes of control (WT) and Syk KD HT (HT) platelets. Mean ± S.D, n ≥ 3. Statistical analysis was performed with a two‐way ANOVA with Bonferroni posttest (ns; *P* > .05). F, Quantification of time to peak of control (WT) and Syk KD HT (HT) platelets. Mean ± S.D, n ≥ 3. Statistical analysis was performed with a two‐way ANOVA with Bonferroni post‐test (ns; *P* > .05, *P < .05). G, Representative time course of Syk Y525/Y526 phosphorylation in WT and Syk KD HT mouse washed platelets following GPVI ligation with 10 µg/mL CRP. Actin acts as a loading control. H, Quantification of control (WT) and Syk KD HT (HT) Syk Y525/Y526 phosphorylation time courses. Mean ± S.D, n = 3. I, Time to maximal Syk Y525/Y526 phosphorylation. Line indicates the mean ± S.D. *P* = .193. Statistical analysis was performed with unpaired *t* test

Modelling was used to determine the sensitivity of Syk phosphorylation at position Y525/526 in response to variation (±50%) of the key components of the Virtual Platelet model (Figure 3). The model predicts that the time to peak Syk phosphorylation at the activatory site in mouse platelets is, unlike in human platelets, insensitive to a 50% change in Syk copy number. In mouse platelets there is no difference in the predicted timing of Syk activation following 50% Syk deficiency predicting that mouse platelets are insensitive to large variations in Syk protein copy number (Figure 3). To test this, using aggregation as a functional endpoint of platelet activation, we used a novel mouse model which expresses a kinase dead (K396R) form of Syk in mice containing a megakaryocyte lineage specific Cre‐deleter, Pf4‐Cre.^39^, ^40^ Platelets express the kinase dead version of Syk and wild‐type Syk at the same level as Syk in control platelets (Figure S1 in supporting information). Heterozygous mice, which have both a wild‐type Syk allele and a K396R kinase dead Syk allele, were compared to litter mate controls (Figure 4A). No significant difference in total Syk protein levels was observed in the Syk KD HT platelets when compared to control platelets (Figure 4B and 4C). Using the assumption that Syk KD HT mice express both wild‐type and kinase dead versions of Syk in a 1:1 ratio we compared the aggregation of platelets from control and Syk KD HT mice in response to 10, 3, and 1 µg/mL of CRP (Figure 4D and 4E). No significant difference in percentage platelet aggregation at 5 minutes following agonist addition was observed between control and Syk KD HT mouse platelets at any of the concentrations of CRP tested (Figure 4E). However, following quantification of the time to peak, a statistically significant delay was seen in the aggregation of Syk KD HT following the addition of 3 µg/mL CRP (Figure 4F). No significant difference in the time to peak was observed following 10 µg/mL CRP.

Tyrosine phosphorylation on the Syk activatory loop was determined by quantitative western blotting (Figure 4G‐I). As the model predicts, no significant difference was seen in the time to maximal tyrosine phosphorylation on the Syk activatory loop between control and Syk KD HT mice following stimulation with 10 µg/mL CRP. In addition, no significant difference was observed in the phosphorylation of the downstream signalling molecule LAT at position Y200 (Figure S2 in supporting information).

Despite the competition of wild‐type and kinase dead versions of Syk in the heterozygous mice and the dominant negative effect this has on signalling outcomes, heterozygous platelets expressing both the wild type and kinase dead version of Syk largely have no observable phenotype when stimulated with 10 µg/mL CRP and only a minor phenotype when stimulated using a reduced concentration of CRP. These data validate the model by demonstrating that mouse platelets are relatively resistant to a 50% variation in functional Syk.

### 3.5 Spatial organization of Syk in mouse and human platelets

**Figure 5:**
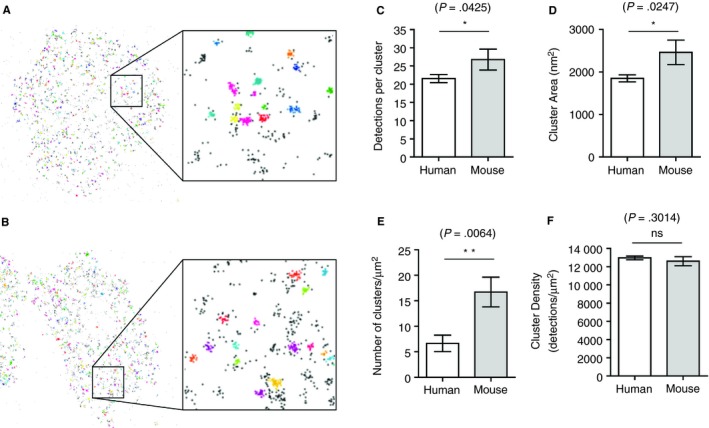
Spatial distribution of Syk in mouse and human platelets. Representative plot of clusters in a human platelet (A) and a mouse platelet (B) spread on CRP. Clusters were identified using DBSCAN with a radius of 25 nm and a minimum number of points as 10. Each cluster is identified by a different color, the black points are background points which do not meet the criteria to be included in a cluster. Quantification of the number of detections per cluster (C), cluster area (D), number of clusters per spread platelet area (E), and the detection density within clusters (F) in human and mouse platelets. Mean ± S.D, n = 3 *P ≤ .05 **P ≤ .01 Statistical analysis was performed with unpaired *t* test

The spatial distribution of receptors and signalling molecules is an important consideration in the regulation of signalling pathways. We hypothesized that as the initial signalling events are similar between mouse and human platelets, both in the Virtual Platelet model and in the experimental outcomes, signalling may be regulated by the spatial distribution of signalling molecules. To identify and quantify the spatial distribution of Syk in human and mouse platelets the localization of Syk was imaged by dSTORM super resolution microscopy using an Alexa 647 conjugated secondary labelled pan‐Syk antibody, which cross reacts with both human and mouse Syk. The DBSCAN algorithm was used to determine Syk cluster size, number, and detection density within clusters (Figure 5A and 5B). DBSCAN identifies clusters by grouping points together within a defined local distance provided a minimum number of points can be found within that defined distance. For this analysis, a radius of 25 nm was used and the minimum number of points located within the defined radius was 10. These user‐defined conditions minimized background noise and minimized the merging of discrete clusters together (Figure S3 in supporting information). While mouse platelets had significantly more detections per cluster, a larger cluster area, and an increased number of clusters per unit area (Figure 5C, 5D, and 5E) there was no significant difference in the Syk detection density within clusters between human and mouse platelets (Figure 5F).

## 4 DISCUSSION

Quantification in biology is of increasing importance, not only to identify potential new drug targets, but to also understand the implications signalling perturbations and variations have on functional outcomes by generating models reliable enough for *in silico* research. Here we use flow cytometry to quantify the level of surface receptors and to quantify changes in the copy number of membrane receptors at the surface of platelets.

All platelet surface receptors tested in this study, except the metalloproteinase ADAM10 and the tetraspanin CD9, were within two‐fold of those determined by quantitative proteomics. The flow cytometry approach for these two proteins gave surface protein copy numbers that were 10 times lower that determined by proteomics. These differences may indicate that these proteins have significant intracellular pools or decreased antibody binding due to steric hindrance. Indeed, studies in other cell types demonstrate that, in addition to surface expression, ADAM10 is also localized to intracellular pools^41^ but it is not known if this is the case in platelets. Tetraspanins are recognized as membrane organizers, interacting with other tetraspanins and also other interacting partners such as integrins.^42^ Clustering of CD9 with other tetraspanins and membrane partners in the cell membrane may lead to reduced antibody binding due to steric hindrance.

While available proteomic datasets provide an estimation of absolute numbers of proteins, these data can be combined with flow cytometry to determine the copy number of proteins expressed at the surface and used to quantify changes in surface expression. For example, as expected, resting mouse platelets have very little P‐selectin molecules at their surface (550 ± 290 molecules per platelet). When activated this increases to 53 538 ± 8875 molecules per platelet. This value is in the same order of magnitude as the total amount of P‐selectin identified in the mouse proteomic dataset (35 970 ± 2712 molecules per platelet). Proteomics provides an indication of total P‐selectin protein copy number suggesting that following platelet activation the majority of P‐selectin is exposed to the surface of platelets.

There were 42 816 ± 637 copies of CLEC‐2 identified on the surface of mouse platelets compared to only 2016 ± 239 copies reported on human platelets.^43^ The copy number of human CLEC‐2 determined by flow cytometry supports the Burkhart et al (24) proteomics dataset, which identified 3700 copies of CLEC‐2 per human platelet. The copy number of mouse CLEC‐2 by flow cytometry strongly supports the mouse proteomic data, which identified 41 652 ± 7759 copies of CLEC‐2 per mouse platelet.^28^ Our data suggest that mouse platelets express approximately 20 times more CLEC‐2 at their surface than human platelets. The increase in protein copy number may contribute to the recent finding that mouse platelets can adhere to and form aggregates on recombinant mouse podoplanin at high shear rates whereas human platelets are unable to do so.^44^, ^45^ The difference in CLEC‐2 copy number raises the suggestion that high levels of Syk in mouse platelets may support other Syk dependent signalling events such as those mediated by CLEC‐2.

When comparing the copy numbers of signalling molecules associated with the GPVI signalling pathway we were surprised to find that there was a 10‐fold increase in Syk molecules in mouse platelets compared to human. Considering how tyrosine kinases play a pivotal role in platelet receptor signal transduction we sought to use the Virtual Platelet model to explore the effect protein copy number may have on GPVI signalling in mouse platelets, particularly in the regulation of Syk. The model corroborated experimental data demonstrating that phosphorylation of the tyrosines within the Syk activatory loop follow a similar time course in mouse and human platelets despite the differences in copy number of Syk.

The Virtual Platelet, a mathematical model of platelet signalling, has allowed the role and importance of specific signalling molecules involved in platelet activation to be determined. We have used the Virtual Platelet to investigate the functional implications of discrepancies between levels of mouse and human proteins in the GPVI signalling pathway. This has shed light on the molecules and processes within platelet cells that regulate GPVI mediated signalling. Models, like the Virtual Platelet, have the potential to aid the development of new anti‐platelet therapies, enable correlations between preclinical toxicity screening and clinical outcomes, and may facilitate personalized therapy.

The Virtual Platelet simulation that used the mouse protein levels predicted a slightly larger difference in Syk temporal regulation compared with the experimental data. This may lie in the subtle difference in the regulation of Tula‐2, a histidine tyrosine phosphatase that negatively regulates Syk, between mouse and human.^46^ Human Tula‐2 has been reported to have a dependence on protein kinase C (PKC). Studies used pan‐inhibitors of PKC suggests that PKC regulation of TULA‐2 negatively regulates Syk activation. Mouse platelets have no reported role for PKC in Tula‐2 regulation.^47^ Mouse platelets may compensate for the loss of this second level of regulation by having greater levels of Tula‐2 compared to human.

The counterintuitive increase in human platelet activity in response to increases or decreases in Syk copy numbers reflects Syk’s tight control of its own activation and inhibition. The copy numbers in human platelets are in balance so that Syk controls its own activation. A mouse model expressing a kinase dead version of Syk was used to investigate the impact a loss of functional Syk had on GPVI mediated signalling. Despite the competition of wild‐type and kinase dead versions of Syk in the heterozygous mice and the dominant negative effect this may have on signalling outcomes, heterozygous platelets expressing both the wild‐type and kinase dead version of Syk largely behaved like control platelets in the experimental conditions tested. The insensitivity of mouse platelets to variation in Syk leads to the hypothesis that in mice, concentrations of Syk may be more tightly controlled upstream, at the cell membrane. Indeed, considerable evidence suggests that the spatial distribution of signalling assemblies is highly regulated. Many membrane receptors do not function as single signalling units but instead associate in multimolecular complexes with the formation of submicron clusters implicated in the initiation, maintenance, and down regulation of signalling pathways.^48^, ^49^, ^50^

GPVI has been shown to oligomerize in response to a range of GPVI ligands in human platelets.^35^ dSTORM allowed fluorophore molecules to be detected and located with high spatial precision. Combined with cluster analysis the relative localization of Syk molecules in human and mouse platelets was determined. Despite the 10‐fold difference in the copy number of Syk in mouse platelets there was only a 2.7‐fold increase in the number of clusters per spread platelet area and a 1.3‐ and 1.2‐fold increase in the cluster area and the number of detections per cluster, respectively. Overall there was no significant difference in the relative density of Syk within the identified clusters. This suggests that regulation of GPVI signalling may be at the level of receptor and explains why, despite large differences in protein copy number, the regulation of GPVI mediated signalling is similar between mouse and human platelets.

In conclusion, we have used a flow cytometry‐based method to validate the available mouse proteomics data, providing a means to quantify mouse platelet surface protein expression and changes induced following platelet activation. Using a systems pharmacology model of human platelet GPVI signalling, the Virtual Platelet, in combination with quantitative flow cytometry and super resolution microscopy, we have identified that, despite large differences in protein copy number, the regulation of proximal signalling events downstream of GPVI is tightly regulated at the level of the cell membrane in both human and mouse platelets.

## CONFLICT OF INTEREST

The authors declare no conflicts of interest.

## AUTHOR CONTRIBUTIONS

J.L.D., A.J.U., A.P.B., and A.Y.P. designed the study, performed experiments, analyzed data, and wrote the paper. Y.D. and T.S. performed experiments. C.P, J.P, E.H., A.H., M.S., and N.S.P. performed experiments and analyzed data. B.N. provided key reagents. All authors reviewed the manuscript.
