# Supplementary figures and images for: Interspecies differences in protein expression do not impact the spatiotemporal regulation of glycoprotein VI mediated activation
Source: J Thromb Haemost. 2019 Dec 6;18(2):485–96. doi: 10.1111/jth.14673 (PMC7027541; doi:10.1111/jth.14673)

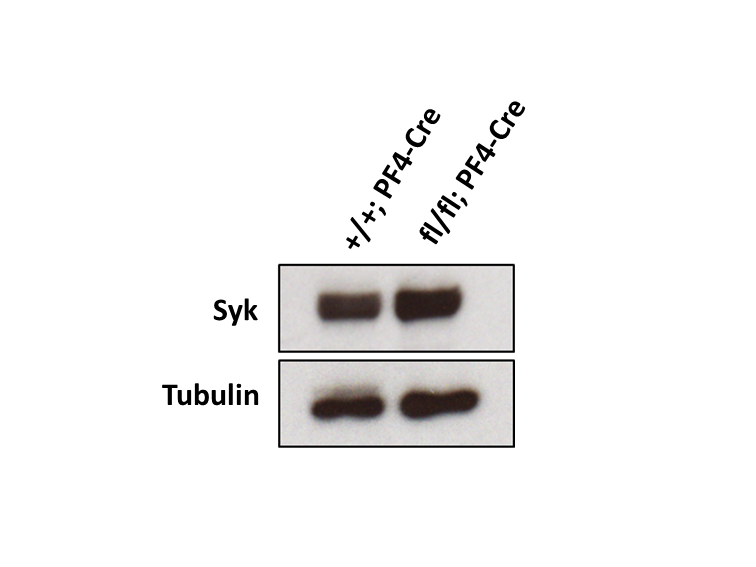

Supplement: Supplementary file 1 [file JTH-18-485-s001.tif]

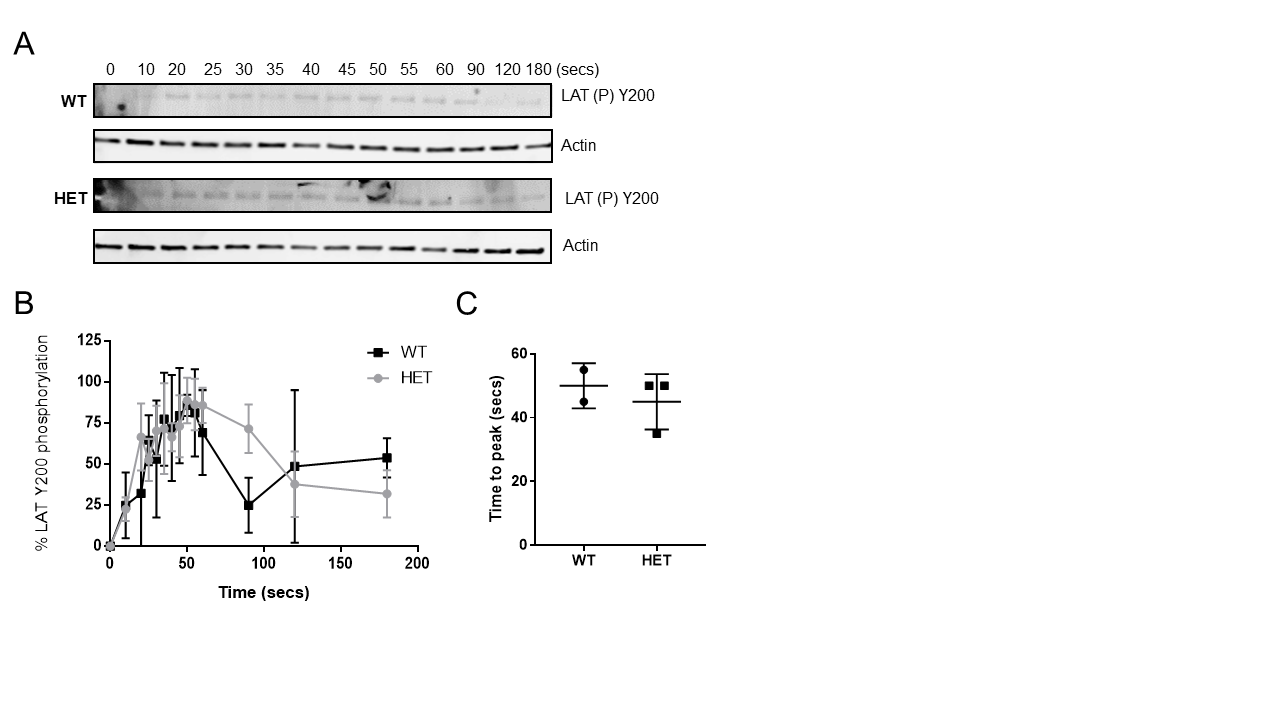

Supplement: Supplementary file 2 [file JTH-18-485-s002.tif]

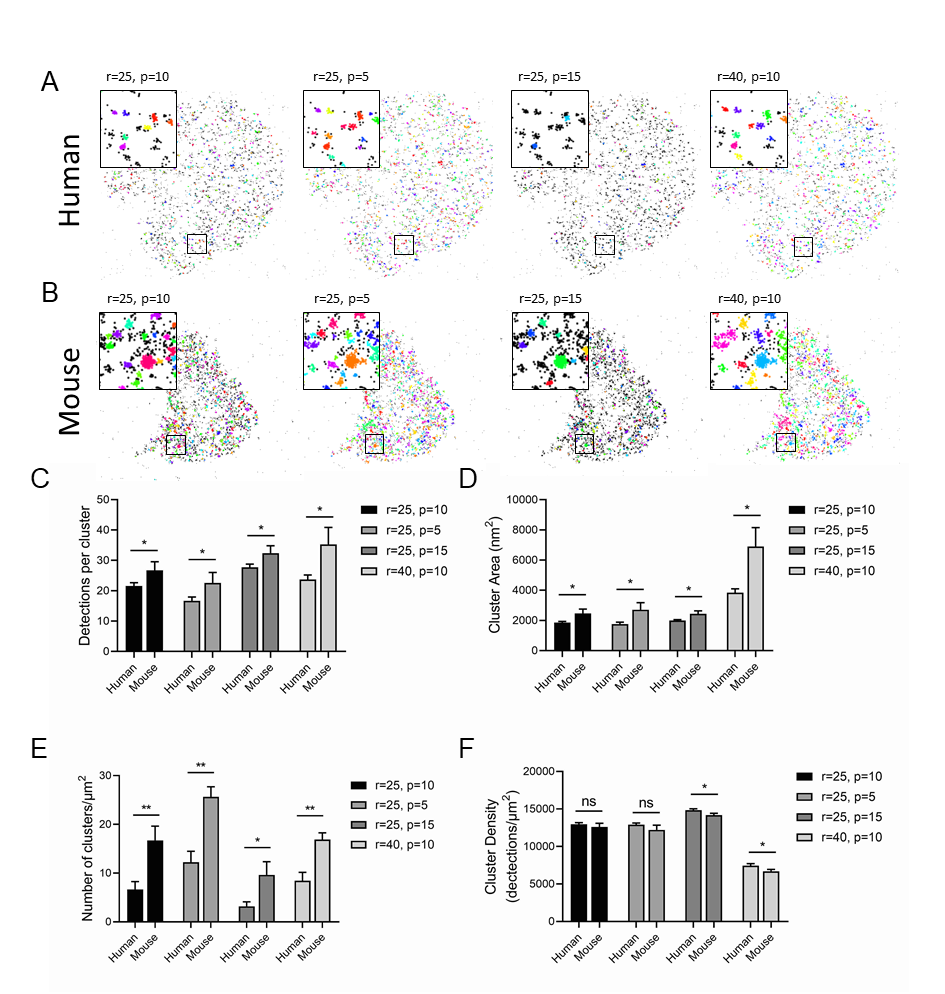

Supplement: Supplementary file 3 [file JTH-18-485-s003.tif]
